# Supplementary material for: Phosphorus Feast and Famine in Cyanobacteria: Is Luxury Uptake of the Nutrient Just a Consequence of Acclimation to Its Shortage?
Source: Cells. 2020 Aug 21;9(9):1933. doi: 10.3390/cells9091933 (PMC7564538; doi:10.3390/cells9091933)
Supplement: Supplementary file 1 [file cells-09-01933-s001.zip › Supplementary methods.pdf]

Supplementary methods

1. Spectral measurements and scattering correction

The OD spectra of the culture samples were taken with the Agilent Cary 300 spectrophotometer equipped with an integrating sphere attachment. Prior to recording of the spectra, the cells were pelleted by centrifugation (3 min, 3000 g), and the cells were re-suspended in the fresh cultivation medium. The supernatant containing, according to the plate count (see above), the bulk of the bacterial cells, was used for the turbidity measurement (at 800 nm). The absorbance of the re-suspended microalgal cells was measured as described below. The correction of the measured OD values for uncertain and variable contribution of scattering by the cells is considered to be essential for accurate monitoring of the microalgal culture growth *via* OD in the long-wave band of Chl absorption [1,2]. The method developed by Merzlyak et al. [2] was used for scattering-correction in this work. This method employs OD in the spectral region unaffected by pigment absorption e.g. in the near infrared to quantify the incomplete collection of light by integrating sphere due to scattering by the sample. Briefly, the spectra of *Nostoc* sp. PCC 7118 cell suspension were recorded in 1 cm glass cuvette at two distances: as close as possible to and 1 cm apart from the entry window of the integrating sphere. In both cases, a cuvette with the cultivation medium was used as a reference. The scattering-corrected optical density at 678 nm then was calculated as

$$OD_{678}^{c} = OD_{678}^{\gamma_I} - \frac{OD_{800}^{\gamma_I}}{OD_{800}^{\gamma_I} - OD_{800}^{\gamma_0}} \times \left[OD_{678}^{\gamma_I} - OD_{678}^{\gamma_0}\right] \quad (1)$$

where OD_{678}^{c} is the scattering-corrected OD_{678}; $OD_{800}^{\gamma_I}$ and $OD_{678}^{\gamma_I}$ are the optical density values at 800 nm (where pigments do not possess detectable absorption) and 678 nm (the long-wave absorption maximum of Chl) measured 1 cm apart from the integrating sphere; $OD_{800}^{\gamma_0}$ and $OD_{678}^{\gamma_0}$ are the optical densities at the same wavelength measured as close as possible to the integrating sphere.

2. Electron microscopy

The microalgae samples for TEM were prepared with the standard protocol: fixed in 2% v/v glutaraldehyde solution in 0.1 M sodium cacodylate buffer (pH 6.8-7.2, depending in the culture pH) at room temperature for 0.5 h and then post-fixed for 4 h in 1% (w/v) OsO_4 in the same buffer. The samples, after dehydration through graded ethanol series including anhydrous ethanol saturated with or without uranyl acetate, were embedded in araldite. Ultrathin sections were made with an LKB-8800 (LKB, Sweden) ultratome, mounted to the formvar coated TEM grids and stained with lead citrate according to Reynolds (1963) and examined under JEM-1011 (JEOL, Tokyo, Japan) microscope.

3. Analytical electron microscopy

The samples for nanoscale elemental analysis in analytical TEM using EDX and EELS were fixed, dehydrated and embedded in araldite or EMbed 812 Resin (Electron Microscopy Sciences, USA) as described above usually excepting the staining with uranyl acetate and lead citrate. Semi-thin and ultrathin sections were made with a LKB-8800 (LKB, Sweden)

ultratome and examined under JEM-2100 (JEOL, Japan) microscope equipped with a LaB_6 gun at the accelerating voltage 200 kV. Point EDX spectra were recorded using JEOL bright-field scanning TEM (STEM) module and X-Max X-ray detector system with ultrathin window capable of analysis of light element starting from boron (Oxford Instruments, UK). The energy range of recorded spectra was 0–10 keV with a resolution of 10 eV per channel. This range includes the peaks of major biogenic elements (C, N, O, P, Ca, Mg, S, K, Na, Cl. At least 10 cells per specimen were analyzed. Spectra were recorded from different parts of electron-dense inclusions (at least 35 measurements for each point) and from other (sub)compartments of microalgae cell (thylakoid membranes, pyrenoid, plastoglobuli, starch grains in chloroplast, mitochondrion, cytoplasmic oil bodies and nucleus). Spectra were processed with INKA software (Oxford instruments, UK) and presented in a range 0.1-4 keV.

EELS analysis was performed on ultrathin sections using Gatan GIF Quantum ER spectrometer (Gatan, USA). EELS point spectra from the vacuolar inclusions were recorded in high-angle annular dark-field scanning transmission electron miscroscopy (HAADF-STEM) mode using Gatan 806 HAADF-STEM detector. The energy–loss range was 100–600 eV, which includes C, N, P, S, Ca, Cl, and O edges. Digital Micrograph software (Gatan, USA) was used for spectra processing. The background was approximated by a power-law function. N and P elemental mapping was carried out using energy-filtered TEM (EFTEM) 3-window method with a 15 eV energy selecting slit. Two pre-edge images at 374 eV, at 392 eV and one post-edge image at 412 eV were recorded for N-mapping, and two pre-edge images at 101 eV, at 123 eV and post-edge image at 150 eV for P mapping. The energy windows were chosen basing on the spectra to avoid overlap with other element edges. The objective aperture was 40 µM. The elemental distributions were calculated automatically using Digital Micrograph software after alignment of the obtained images using a power law as the background model. Specimen thickness was controlled to ensure that it did not exceed the optimal value for EFTEM (below 0.5 λ, where λ is inelastic mean free pathlength for the chosen experimental conditions).

4. Cell morphometry

The quantitative morphometric analyses was carried out using an approach previously developed in our laboratory (Gorelova *et al.*, 2015b). Briefly, the organelles were counted on the sections through the cell equator or subequator (at least ten samples from each treatment were examined). Frequencies of the structures were calculated as total section number percentage of cell sections containing the structure of interest. Linear sizes (cell wall, CW, and its layer thickness, as well as thickness of the appressed membrane pairs and the lumen width) were measured on the TEM micrographs of the cell ultrathin sections (n = 20) using ImageJ software (NIH, Bethesda MA, USA). The significance of the mean difference was tested with Student's t test using Origin software (OriginLab, Northampton MA, USA).

5. DAPI staining

1 µl of 300 µM solution of 4',6-diamidino-2-phenylindole (DAPI) in methanol prepared from a 5 mg/ml stock DAPI for nucleic acid (Sigma, USA) staining in methanol was added (300 nM final concentration) to cell suspension in corresponding media. After 5 min of incubation, aliquots of the cell suspension were transferred in a 96-well transparent plate. The plate was read on the plate reader Infinite 200 Pro (Tecan, Switzerland) in the bottom-read mode, and DAPI fluorescence was measured using UV excitation (360 nm). The fluorescence spectra were recorded in the 450–645 nm and processed by Magellan (Tecan, Switzerland)

software. The fluorescence spectra of the same media without cells were used to subtract the background signal. The spectra were measured every 10 m for 4 h after the DAPI addition.

References

1. Merzlyak, M.; Chivkunova, O.; Maslova, I.; Naqvi, K.; Solovchenko, A.; Klyachko-Gurvich, G. Light absorption and scattering by cell suspensions of some cyanobacteria and microalgae. *Russ. J. Plant Physiol.* **2008**, *55*, 420-425, doi:10.1134/S1021443708030199.
2. Merzlyak, M.N.; Naqvi, K.R. On recording the true absorption spectrum and the scattering spectrum of a turbid sample: application to cell suspensions of the cyanobacterium *Anabaena variabilis*. *J. Photochem. Photobiol. B: Biol.* **2000**, *58*, 123-129, doi:10.1016/S1011-1344(00)00114-7.
