# Supplementary material for: Phosphorus Feast and Famine in Cyanobacteria: Is Luxury Uptake of the Nutrient Just a Consequence of Acclimation to Its Shortage?
Source: Cells. 2020 Aug 21;9(9):1933. doi: 10.3390/cells9091933 (PMC7564538; doi:10.3390/cells9091933)
Supplement: Supplementary file 1 [file cells-09-01933-s001.zip › Solovchenko_LPU_supplemental.pdf]

Supplementary material

Table S1. Primers used for qRT-PCR in this work.

Inorganic pyrophosphatase (*ppa*; all3570)	ppaRT fwd	GCTACGCTCACGTAAAATCTTTG
	ppaRT fwd	TTTGTAACTACGGAAAAACTCAGC
P_i transporter (*pstS1*; all4575)	pstS1RT fwd	ACTAGCTTACAACTTGCCTGG
	pstS1RT rev	AGGTTGACATTAGGGTTAGCAG
P_i transporter (*pstS2*; all0911)	pstS2RT fwd	GATGACGGATGCTGAAATTGC
	pstS2RT rev	TGTTGCACGGGATAATCTGAG
Phosphatase/kinase 2 (*ppk2*; all2088)	ppk2RT_fwd	GTAAATGATGTGCGTGAAAGGG
	ppk2RT_rev	GTGTAAACCATTTGCGATCGG
Polyphosphate kinase 1 (*ppk1*; alr3593)	ppk1RT_fwd	TCGTCTATGGTTTAGTGGGTCT
	ppk1RT_rev	CTGTATAGAGTCGTGCCGTTT
Polyphosphate-dependent glucokinase (*ppgk*; all1371)	PPGKRT_fwd	AAAGGTAAGGGTGTAGAGTTGG
	PPGKRT_rev	CTTCGTAAGTTTCCCCTTTGC
Exo-polyphosphatase (ppx; all3552)	ppxRT_fwd	GAGAATGACAAATGCCGAAAGAG
	ppxRT_rev	AATGACTCCACACCCAACAG
Inositol monophophatase (all2917)	vtcRT_fwd	TTTGGGTAAGTTAGAGGACGC
	vtcRT_rev	TGGAGTGTTGAGGAAAGTGAC
Ribonuclease (*rnpA*) — endogenous control	rnpART_fwd	GCGAGCATTAAAACCGTCATC
	rnpART_rev	ACCGCCCTCTTACTAACTTTG

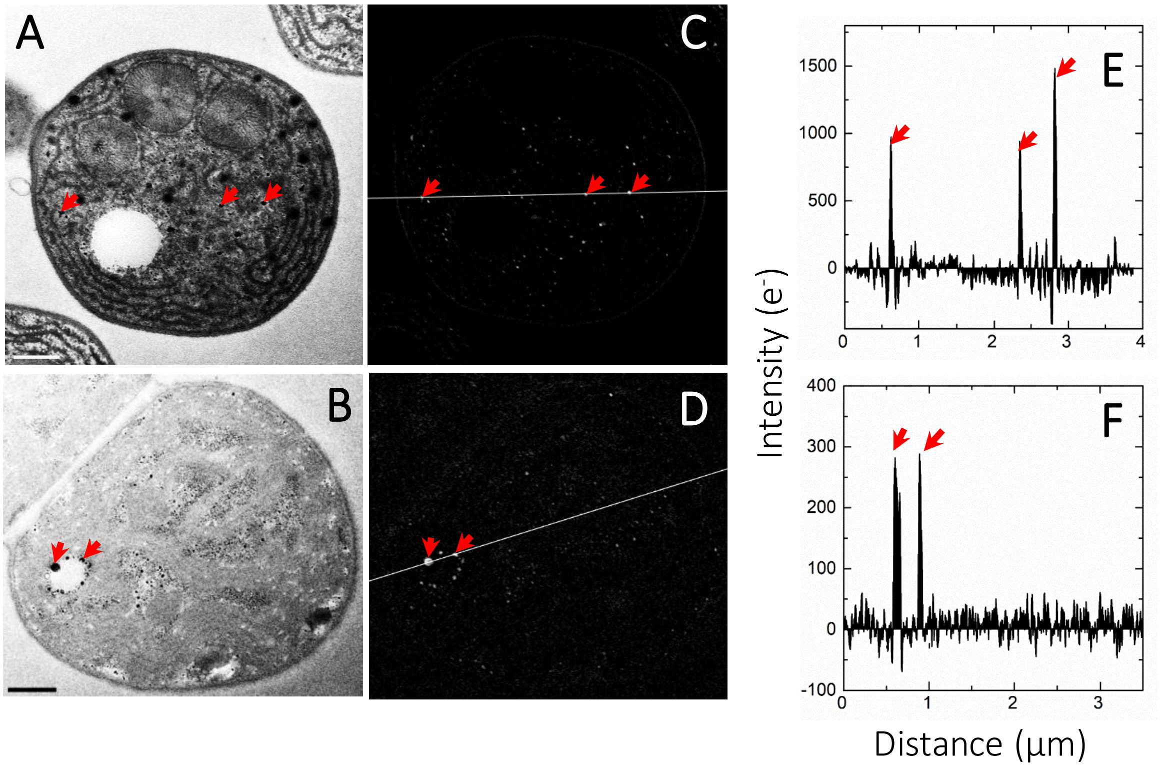

Fig. S1. Accumulation of polyphosphate in the cells of *Nostoc* sp. PCC 7118 visualized energy-filtered transmission electron microscopy (EFTEM). Transient polyphosphate accumulation during lag-phase (4 h after re-feeding of P-starved cells with P_i; A, C, E) and re-accumulation of polyphosphate at early stationary phase (7 d after re-feeding of P-starved cells with P_i; B, D, F; see also Figs. 6, 7). TEM images (A, B) along with the corresponding EFTEM P-maps (C, D), and P signal intensity profiles (E, F, see the white lines in C, D) are shown. Scale bar = 0.5 µm.

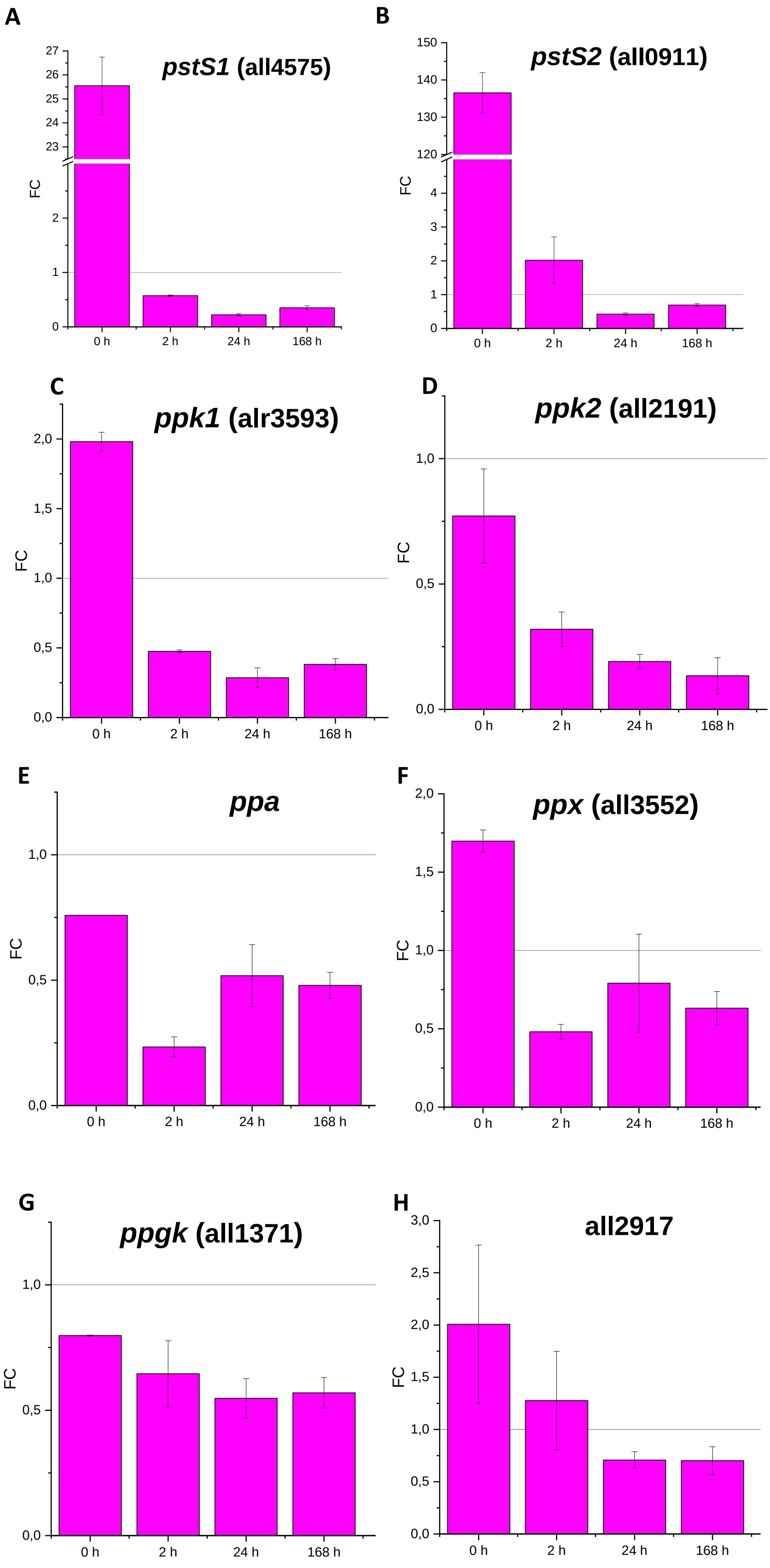
A
pstS1 (all4575)
FC
0 h
2 h
24 h
168 h
B
pstS2 (all0911)
FC
0 h
2 h
24 h
168 h
C
ppk1 (alr3593)
FC
0 h
2 h
24 h
168 h
D
ppk2 (all2191)
FC
0 h
2 h
24 h
168 h
E
ppa
FC
0 h
2 h
24 h
168 h
F
ppx (all3552)
FC
0 h
2 h
24 h
168 h
G
ppgk (all1371)
FC
0 h
2 h
24 h
168 h
H
all2917
FC
0 h
2 h
24 h
168 h

Fig. S2. Changes in the expression of the genes coding for the (A, B) P_i transporters; (C, D) putative polyphosphate kinases; (E) inorganic pyrophosphatase; (F) endonuclease; (G) polyP-dependent glucokinase; and (H) an inositol monophophatase in the P-starved cells *Nostoc* sp. PCC 7118 at different times after Pi re-feeding (indicated on the graphs) estimated with qRT-PCR (see Methods). The transcript levels of the target genes vs. ribonuclease rnpA (endogenous control) were calculated on fold-change scale relative to those at P-sufficient logarithmic growth.

Table S2. Changes in the expression level (log_2FC FPKM calculated relative to the P-sufficient exponentially growing preculture) of the genes involved in response to shortage of P and, potentially, in its luxury uptake in *Nostoc* sp. PCC 7118. The tentative functional annotation was given according to the published genome of its parent strain *Nostoc* sp. PCC 7120 [1-4].

ORF	Name (description)	Conditions			
		Preculture (+P)	P-starved (–P)	Recovery (+P)	
				1 day	7 days
all4502	***sphS*** (P-starvation response regulator, sensory histidine kinase)	0.28	-0.97	-0.47	-0.62
all4503	***sphR*** (P-starvation response regulator)	0.53	-1.39	-0.82	-0.81
all4501	***phoU*** (P-starvation response regulator, repressor of *Pho* regulon)	-0.14	-0.6	-0.52	0.46
all4573	***pstA1*** (ABC-type P_i transporter/binding protein)*	-1.92	-0.43	-0.47	-0.1
all4572	***pstB1*** (ABC-type P_i transporter/binding protein)	-1.56	-0.28	-0.1	-0.58
all4574	***pstC1*** (ABC-type P_i transporter/binding protein)*	-2.76	-0.13	-0.29	-0.09
all4575	***pstS1*** (ABC-type P_i transporter/binding protein)*	-3.74	-0.18	-0.37	-0.69
alr1094	***sphX*** (putative periplasmic P_i transporter – P_i-binding protein, ABC-type)	-0.46	-0.56	-0.65	0.06
all0917	putative periplasmic transporter, ABC-type	-5.43	1.8	0.96	0.75
all0907	***pstX*** (an ABC-type P_i transporter/binding protein)*	-1.01	0.03	-1.09	-0.59
all0909	***pstA2*** (ABC-type P_i transporter/binding protein)*	-2.76	0.88	-0.16	-1.92
all0908	***pstB2*** (ABC-type P_i transporter/binding protein)*	-0.94	-0.4	-0.9	-0.45
all0910	***pstC2*** (ABC-type P_i transporter/binding protein)*	-3.62	-0.37	-0.58	-0.79
all0911	***pstS2*** (ABC-type P_i transporter/binding protein)*	-6.09	0.95	-0.03	-0.78

*High-affinity P_i transporters residing in the cytoplasmic membane.

Table S3. Changes in the expression level (log_2FC FPKM calculated relative to the P-sufficient exponentially growing preculture) of the genes involved in phosphonate uptake and assimilation in *Nostoc* sp. PCC 7118. The tentative functional annotation ois given according to the published genome of its parent strain *Nostoc* sp. PCC 7120 [1-3].

ORF	Name	Conditions			
		Preculture (+P)	P-starved (–P)	Recovery (+P) 1 day	Recovery (+P) 7 days
all2230	***phnC***	-4.74	0.38	-1	-1
all2228	***phnD***	-2.53	-1.38	-1.8	-1
alr2226	***phnE***	-2.85	0.25	0.11	0.77
alll2225	***phnG***	-0.39	-0.99	-5.38	-1.25
all2224	***phnH***	-0.66	0.48	-1.38	-1.38

Table S4. Changes in the expression level (log_2FC FPKM calculated relative to the P-sufficient exponentially growing preculture) of the genes encoding the components of the light-harvesting antenna and RuBisCo in *Nostoc* sp. PCC 7118. The tentative functional annotation ois given according to the published genome of its parent strain *Nostoc* sp. PCC 7120 [4].

Name (description)	Conditions			
	Preculture (+P)	P-starved (–P)	Recovery (+P)	
			1 day	7 days
pecA (phycoerythrocyanin alpha chain)	4.26	-9.41	-5.21	-0.99
pecB (phycoerythrocyanin beta chain)	4.23	-10.61	-4.79	-1.09
pecC (phycoerythrocyanin-associated rod linker protein)	4.54	-7.71	-4.77	-1.67
pecE (bilin biosynthesis protein)	4.62	-6.25	-4.76	-2.52
**pecF** (bilin biosynthesis protein)	3.25	-4.69	-3.4	-2.61
cpcA (phycocyanin alpha chain)	2.48	-5.2	-0.57	0.73
cpcB (phycocyanin beta chain)	2.49	-5.35	-0.58	0.7
cpcC (phycocyanin-associated rod linker protein)	3.53	-5.38	-1.61	0.27
cpcD (rod-capping linker polypeptide)	3.87	-7.34	-1.44	0.54
cpcE (phycocyanobilin lyase alpha subunit)	4.26	-7.84	-1.67	0.62
cpcF (phycocyanobilin lyase beta subunit)	3.86	-6.56	-1.52	-0.02
cpcG1 (phycobilisome rod-core linker protein)	3.37	-5.61	-1.17	0.1
cpcG2 (phycobilisome rod-core linker protein)	3.24	-4.01	-1.14	0.22
cpcG3 (phycobilisome rod-core linker protein)	3.24	-4.35	-1	0.07
cpcG4 (phycobilisome rod-core linker protein)	0.79	-1.41	0.21	0.69
rbcL (RuBisCo large subunit)	1.35	-1.25	-0.08	-0.53
rbcS (RuBisCo small subunit)	1.34	-1.33	0.07	-0.46
rca (RuBisCo activase)	-0.35	-0.79	0.41	-0.69

Table S5. Changes in the expression level (log_2FC FPKM calculated relative to the P-sufficient exponentially growing preculture) of the genes encoding the enzymes of cyanophycin turnover in *Nostoc* sp. PCC 7118. The tentative functional annotation ois given according to the published genome of its parent strain *Nostoc* sp. PCC 7120 [4].

ORF	Name (description)	Conditions			
		Preculture (+P)	P-starved (–P)	Recovery (+P)	
				1 day	7 days
–	***cphA1*** (cyanophycin synthase)	-1.82	1.09	0.53	0.85
–	***cphA2*** (cyanophycin synthase)	0.24	-0.19	0.08	0.08
–	***cphB2*** (cyanophycin synthase)	1.11	-1.03	-0.34	-0.11
all3922	– (isoaspartyl dipeptidase)	-0.43	1.27	0.75	0.13

References

1. Su, Z.; Olman, V.; Xu, Y. Computational prediction of Pho regulons in cyanobacteria. *BMC Genomics* **2007**, *8*, 156, doi:10.1186/1471-2164-8-156.
2. Ault-Riché, D.; Fraley, C.D.; Tzeng, C.-M.; Kornberg, A. Novel assay reveals multiple pathways regulating stress-induced accumulations of inorganic polyphosphate in Escherichia coli. *Journal of bacteriology* **1998**, *180*, 1841-1847.
3. Suzuki, S.; Ferjani, A.; Suzuki, I.; Murata, N. The SphS-SphR Two Component System Is the Exclusive Sensor for the Induction of Gene Expression in Response to Phosphate Limitation in Synechocystis. *Journal of Biological Chemistry* **2004**, *279*, 13234-13240, doi:10.1074/jbc.M313358200.
4. Kaneko, T. Complete Genomic Sequence of the Filamentous Nitrogen-fixing Cyanobacterium Anabaena sp. Strain PCC 7120. *DNA Research* **2001**, *8*, 205–213.
